# A haptics-assisted cranio-maxillofacial surgery planning system for restoring skeletal anatomy in complex trauma cases

**DOI:** 10.1007/s11548-013-0827-5

**Published:** 2013-04-21

**Authors:** Pontus Olsson, Fredrik Nysjö, Jan-Michaél Hirsch, Ingrid B. Carlbom

**Affiliations:** 1 Centre for Image Analysis, Uppsala University, Uppsala, Sweden; 2 Department of Surgical Sciences, Oral and Maxillofacial Surgery, Uppsala University, Uppsala, Sweden

**Keywords:** Cranio-maxillofacial surgery planning, Haptics, Stereo visualization, Collision detection, Snap-to-fit

## Abstract

**Purpose:**

Cranio-maxillofacial (CMF) surgery to restore normal skeletal anatomy in patients with serious trauma to the face can be both complex and time-consuming. But it is generally accepted that careful pre-operative planning leads to a better outcome with a higher degree of function and reduced morbidity in addition to reduced time in the operating room. However, today’s surgery planning systems are primitive, relying mostly on the user’s ability to plan complex tasks with a two-dimensional graphical interface.

**Methods:**

A system for planning the restoration of skeletal anatomy in facial trauma patients using a virtual model derived from patient-specific CT data. The system combines stereo visualization with six degrees-of-freedom, high-fidelity haptic feedback that enables analysis, planning, and preoperative testing of alternative solutions for restoring bone fragments to their proper positions. The stereo display provides accurate visual spatial perception, and the haptics system provides intuitive haptic feedback when bone fragments are in contact as well as six degrees-of-freedom attraction forces for precise bone fragment alignment.

**Results:**

A senior surgeon without prior experience of the system received 45 min of system training. Following the training session, he completed a virtual reconstruction in 22 min of a complex mandibular fracture with an adequately reduced result.

**Conclusion:**

Preliminary testing with one surgeon indicates that our surgery planning system, which combines stereo visualization with sophisticated haptics, has the potential to become a powerful tool for CMF surgery planning. With little training, it allows a surgeon to complete a complex plan in a short amount of time.

## Introduction and related work

One fundamental task in cranio-maxillofacial (CMF) surgery is to restore normal skeletal anatomy in patients with extensive fractures of the facial skeleton and mandible from gunshot wounds, work-related injuries, natural disasters, or traffic accidents. Any attempt to restore a bone fragment to its original position poses considerable risk for additional damage to vital anatomical structures. Furthermore, small errors in the positioning of each fragment may accumulate and result in inadequate reconstruction which in turn may result in poor function and a poor esthetic result.

Surgical planning from CT data may improve the surgical outcome and reduce the time in the operating room. But if the planning relies only on visual cues, object contact and object penetration can be difficult to discern because contact surfaces are likely to be occluded by the many bone fragments. Current commercially available CMF surgery planning systems, for example systems by Planmeca [1], Materialise [2], and Brainlab [3], rely primarily on two-dimensional graphical interfaces. These put great demands on the user, requiring that he or she be able to visualize complex 3D models from their 2D projections on a two-dimensional display and be able to plan delicate 3D procedures using a set of 2D projections and 2D interaction tools. Furthermore, while a surgeon relies heavily on his/her sense of touch in the operation room, surgical planning systems generally do not use the sense of touch to complement the visual interface. As a result, CMF surgery planning is time-consuming and cumbersome, making it difficult to find an optimal surgical strategy, which in turn may result in a less than perfect reconstruction with needless patient discomfort and loss of functionality.

Several research groups have developed systems for CMF surgery planning. Essig et al. [4] and Rana et al. [5] describe interactive computer-assisted planning and surgery tools using photorealistic imaging for optimized treatment of oral and maxillofacial malignancies, and for tissue engineering of bone.

Juergens et al. [6, 7] describe planning tools that include skull and soft tissue segmentation, assessment of skeletal muscle properties, characterization of the mechanical response of soft facial tissue, clinical validation, and transfer of the CMF planning into the operating room. However, haptics was not explored as an interaction modality in these systems.

Haptics has the potential to improve surgical planning by giving the surgeon virtual tools that are familiar from the operating room: s/he can *feel* if two bone fragments fit together or if the occlusion (bite) is correct. Contact forces also help the surgeon to avoid interpenetration of fragments that may be difficult to discern visually. Forsslund et al. [8] present a requirements study for CMF surgery planning with haptic interaction for bone fragment and plate alignment, exploring what features might be important in haptic cranio-maxillofacial planning. This is done with physical mock-ups, complemented by the implementation of some features in software. They mention “haptic fidelity” as a highly important aspect for success in this type of system.

Haptic feedback is used to increase the realism in simulators for training of specific surgical procedures. Pettersson et al. [9] present a simulator for cervical hip fracture surgery training which provides visuo-haptic feedback of the drilling task central to this procedure. Morris et al. [10] describe a bone surgery training simulator also with focus on drilling, in this case of the temporal bone and the mandible. This last simulator provides audio feedback in addition to the visual and haptic feedback. A survey of visuo-haptic systems for surgical training with a focus on laparoscopic surgery can be found in [11].

We present a system that combines stereoscopic 3D visualization with six-DOF haptic rendering that can be used by a surgeon with only minimal training. The system features a head tracker to enable user “look-around” in the graphical scene, a simulated spring coupling between the manipulated virtual bone fragment and the haptic handle for enhanced haptic stability, high-precision collision detection, the ability to group and manipulate a set of fragments as one entity, and Snap-to-fit, a tool for precision alignment of matching bone fragments.

## Methods

### System overview

**Fig. 1:**
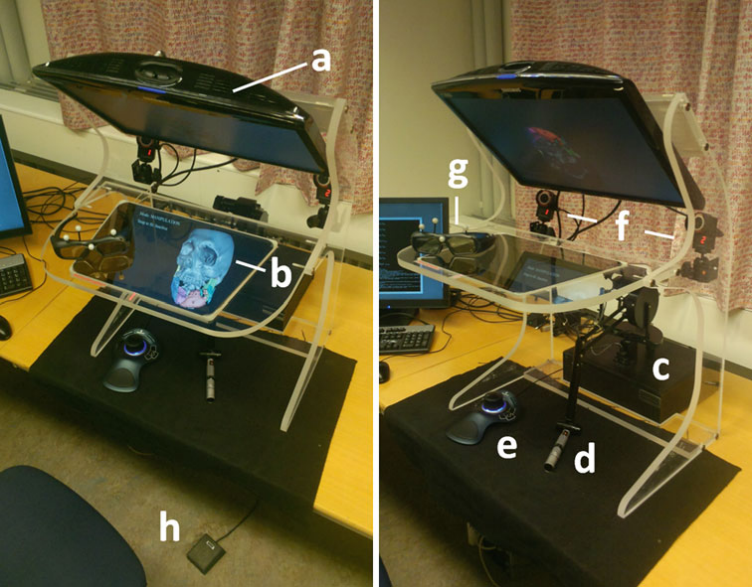
The planning system hardware as seen from above (*left*) and from the side (*right*). The graphical objects are displayed by the monitor (**a**) and reflected on the half-transparent mirror (**b**). The user manipulates the 3D graphical objects with the Phantom Premium device (**c**) using its handle (**d**) under the half-transparent mirror. The pushbuttons on the 3DConnexion controller (**e**) under the mirror activate the grouping tool and Snap-to-fit fragment surface marking. The two infra-red cameras (**f**) mounted under the display track the marker rig mounted on the shutter glasses (**g**) for user “look-around.” The foot-switch (**h**) activates the Snap-to-fit attraction forces

The patient data comprise segmented volumetric CT data from the fractured regions in which independent bone fragments are labeled. (See section “Image data handling.”) A half-transparent mirror with stereo glasses gives the user a stereoscopic view of the data, and the haptic unit, positioned under the mirror, has a handle for moving the entire CT model or individual bone fragments. (See Fig. 1.)

A head tracker, which continually updates the user’s vantage point, gives the user “look-around,” that is the ability to view objects from different angles by simply moving his/her head. This is essential for detecting bone fragments that may be (partially) occluded from certain vantage points.

**Fig. 2:**
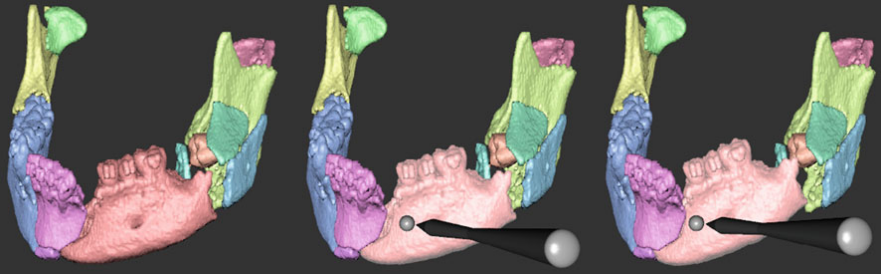
Virtual reconstruction of a mandible. Each individual bone fragment is given a *unique color* (*left*). When the haptic cursor is held close to a bone fragment, it is *highlighted* (*middle*) and the user can then grasp and manipulate it with the six-DOF haptic handle (*right*). Contact forces guide the user during manipulation

Each segmented bone fragment is assigned a unique color for clear visual identification. A haptic cursor follows the six-DOF motions of the haptic handle in the visual space. When the haptic cursor is in close proximity to a bone fragment (or group of fragments), the fragment or group is graphically highlighted indicating that the user may pick it up and manipulate it (affect its position and rotation) by moving the handle with the handle button depressed. (See Fig. 2.) When the haptic cursor is further away from the bone fragments, and no fragment is highlighted, the user may translate and rotate the entire 3D volume, again by moving the handle with its button depressed.

During fragment manipulation, contact force and torque from contacts with other fragments are rendered haptically with high spatial resolution, giving the user an impression similar to that of manipulating a real, physical object around other objects. To limit inter-object penetrations, we simulate a translational and a rotational spring, commonly known as virtual coupling, between the bone fragment currently under manipulation and the haptic handle. The user may push a manipulated bone fragment toward another bone fragment which stretches the simulated spring, but the manipulated fragment stops at the other fragment’s surface instead of penetrating it. This increases the stability of the haptic interaction dramatically [12].

When two or more fragments have been positioned relative to one another, the user may group them and manipulate them as one unit. Additional fragments may subsequently be attached to extend the group and they may also be detached from the group. When bone fragments are grouped, the entire group is given one color. The grouping tool is activated with pushbuttons on the 3DConnexion unit placed to the left under the half-transparent mirror. (See Fig. 1.)

In what follows, we describe in more detail the unique feature Snap-to-fit, which complements the contact forces to aid the user in bone fragment alignment.

### Snap-to-fit, a complement to contact forces

**Fig. 3:**
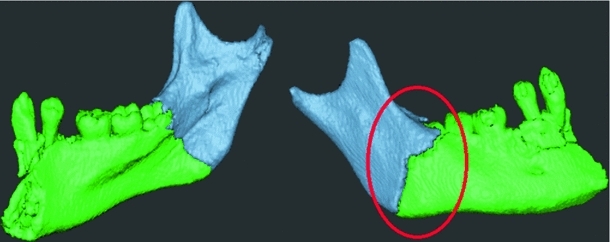
Alignment of two mandibular fragments using contact forces alone, without Snap-to-fit. Visually, the fit may look acceptable from one viewpoint (*left*), but when seen from the opposite side (*right*), it is clear that the alignment is sub-optimal. Haptic contact forces provide some guidance, but depending on the quality of the haptic display it may still be difficult to feel the optimal fit

Precise alignment of the bone fragments is important since even small rotational and translational errors between fragments may accumulate to much larger errors as a result of the reconstruction of a series of fragments, for example, a mandible with multiple fractures. But due to visual occlusion, it may be difficult to visually discern the ideal fit between two bone fragments. Just as we use our human haptic ability in the real world to assemble a broken object, six-DOF contact forces may provide haptic guidance in finding an optimal fit between two fragments. However, limited force fidelity in most commercial haptic devices of today makes it difficult to feel when the optimal fit is found as clearly as can be done with real, physical objects. (See Fig. 3.)

**Fig. 4:**
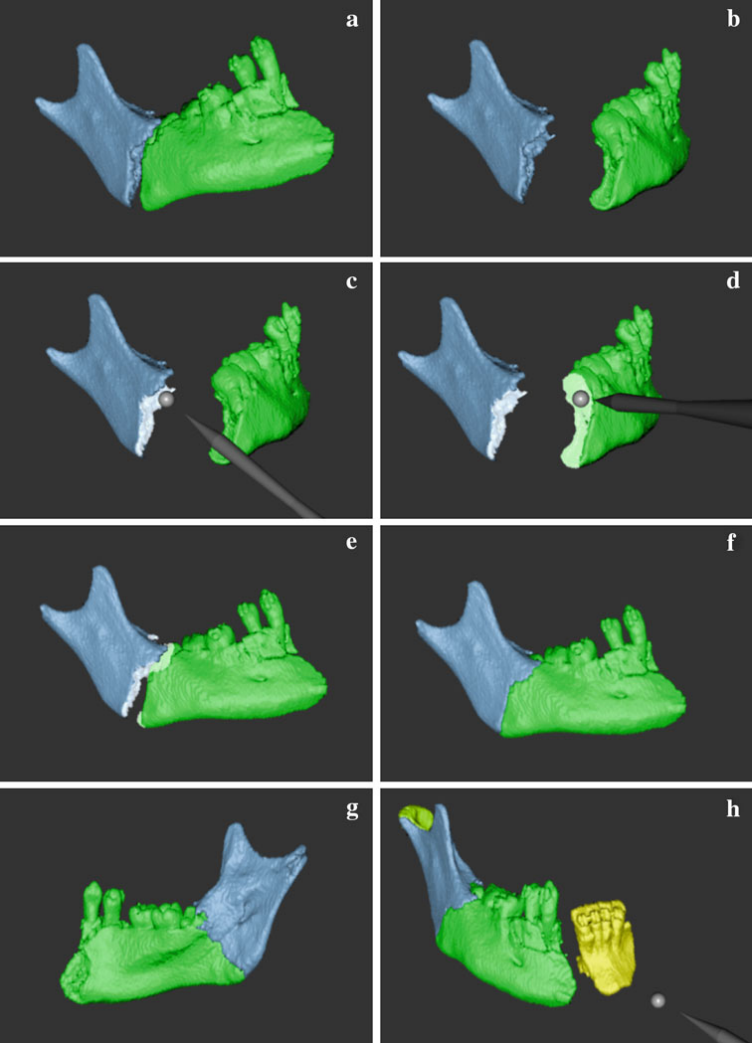
Alignment of two fragments of a mandible using Snap-to-fit with fracture surface marking. Two fragments to be aligned (**a**). Fragments oriented so that the fracture surfaces are visible (**b**). The user marks the fracture surfaces using the haptic cursor as a paintbrush (**c**, **d**). The fragments are coarsely aligned (**e**). The user activates Snap-to-fit (**f**). Fragments seen from the opposite side (**g**). Additional fragments are aligned to continue the reconstruction of the mandible (h)

The alignment tool, Snap-to-fit, complements haptic contact forces in search for a good fit between two bone fragments. For a detailed description of Snap-to-fit, we refer the reader to [13]. In summary, the user begins by moving a bone fragment close to a matching fracture surface on another bone fragment. From this approximate initial position of the two fragments, the user activates Snap-to-fit with the foot-switch (shown in Fig. 1) that engages attraction forces computed from the fracture surfaces. The forces pull the manipulated objects toward the closest stable fit, that is, it “snaps” the fragments to a local stable fit (see Fig. 4). We scale the attraction forces by the similarity of the fracture surfaces, computed by the colinearity of the surface normals. Fragments with matching surfaces have stronger attraction forces than those with less similar surfaces.

Snap-to-fit works best when the fracture surfaces of both fragments are well preserved by the segmentation and the fragments are not too thin or too small. For some types of fractures, for example, compression fractures, the fracture surface may be damaged with portions of the surface missing. In these cases, a good match between the fracture surfaces may not be possible and the user has to override the system and use his/her expertise to manually find a suitable placement of the bone fragments.

One limitation in the original implementation of Snap-to-fit is that the fragments may “snap” to several alternative positions, since the whole fragment surface is a potential matching surface. We therefore extend the method described in [13] with the ability to mark portions of fragment surfaces as fracture surfaces, allowing the user to “paint” the fracture surfaces with the haptic cursor (see Fig. 4). Only painted surfaces are included in the attraction force model, which prevents the fragment from snapping to false regions outside the fragment surface area.

### Hardware and implementation details

Our planning system executes on an HP Z400 Workstation with an Nvidia Quadro 4000 Graphics Processing Unit (GPU) driving a Samsung 120 Hz stereo monitor which displays time-multiplexed stereo graphics at a resolution of $1,680\times 1,050$ synchronized with a pair of Nvidia 3D Vision Pro [14] shutter glasses. The half-transparent mirror rig used for visuo-haptic collocation is manufactured by SenseGraphics [15]. The head tracker is based on an IR optical tracker (OptiTrack from Natural Point [16]), with built-in motion capture and image processing, that optically tracks a marker rig consisting of four IR-reflecting spherical markers placed asymmetrically on the stereo glasses worn by the user. After careful registration of the tracking frame with the visual frame, the head tracker estimates the user vantage point from which we render the stereo perspective.

We render the bone fragment surfaces on the visual display using splatting [17] which is implemented on the GPU to achieve real-time rendering.

We use a Sensable Phantom Premium 1.5 High Force/6DOF haptic device [18], with six-DOF (in/out) running at a haptic frame rate of 1 kHz. We render the six-DOF contact forces using a rigid body contact model combined with a virtual spring (static virtual coupling) which decouples the manipulated bone fragment position and orientation from the haptic handle to improve the haptic stability [12]. We rely heavily on pre-computation and hierarchical data-structures in order to achieve real-time haptic interaction rates [13]. The contact force model and the static virtual coupling are detailed in [19]. Snap-to-fit is implemented according to [13] with the extension that the user may mark fracture surface areas. Only marked surface areas are included in the attraction force model.

### Image data handling

We load the patient-specific volumetric image data from a DICOM stack of CT images. The images in this study have a resolution of 0.35 mm, and the inter-slice distance is 0.60 mm. We first segment bone tissue from soft tissue by thresholding the CT data and then remove small, isolated tissue components with fewer than 100 connected voxels using the *bwareaopen *filter in MATLAB. We manually segment and label individual bone fragments from the resulting image volume using ITK-Snap [20] before loading them into the planning system.

## System evaluation

**Fig. 5:**
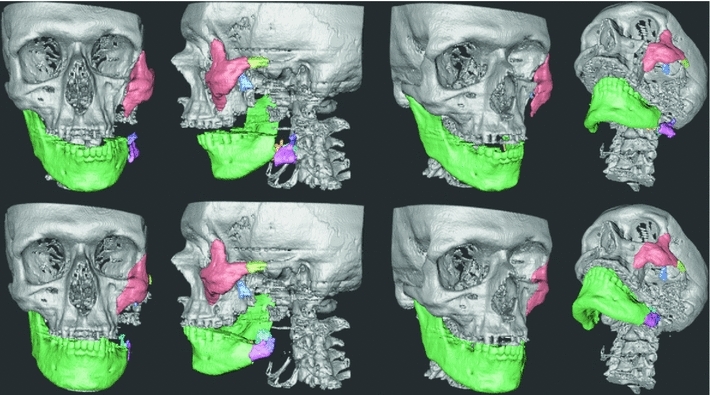
Practice case pre-planning (*first row*) and after the completed virtual restoration (*second row*). Snap-to-fit was used to position the zygomatic bone (*red fragment*) into its correct place

We invited an experienced CMF surgeon, who did not have any prior experience with our system, to plan two trauma cases. He first received 45 min of training, which consisted of planning the reconstruction of a patient with the facial fractures shown in Fig. 5; we denote this case as the *Practice case*. During the practice planning, we gave oral instructions of how to use the haptic device, head tracker, all features of the system, and supported the surgeon in the planning process. When the training was completed, we asked him to complete, on his own, a plan of the complex trauma case denoted the *Evaluation Case*. In the evaluation case, we focused on the reconstruction of the mandible and disregarded some additional fractures, such as the zygomatic bone and orbit on the left hand side, which should be addressed in a complete plan.

### Practice case

A 59-year-old male had sustained major trauma to his left maxillofacial region resulting in a major defect of the horizontal and vertical part of the mandible as well as a fracture of the zygomatic bone with moderate displacement of the total zygomatic complex without any fragmentation. (See Fig. 5.) The training session involved using the features of the system, including Snap-to-fit, in order to position the zygomatic complex correctly. To position the zygoma, the surgeon marked the full fracture surface on the zygomatic bone and its corresponding fracture surface on the cranium. He then moved the bone fragment into an approximate initial position and activated Snap-to-fit which produced the result shown in Fig. 5.

### Evaluation case

**Fig. 6:**
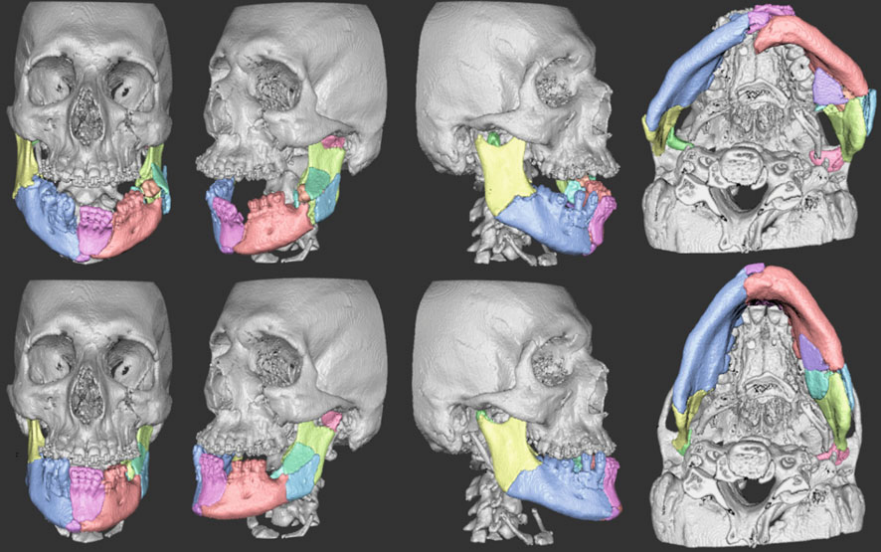
Evaluation case pre-planning (*first row*) and after the completed virtual restoration (*second row*). All mandibular fractures are adequately reduced. The occlusion is not optimal due to interference from dislocated teeth (44, 43) which also led to the anterior position of the temporomandibular joints in the joint space

An 18-year-old male fell from a sky lift 16 m and sustained comminute fractures of the skull, midface, and mandible. The parasymphyseal region of the mandible exhibited a complex fracture with broad diastasis, small interpositioned bone fragments, and overlapping bone fragments. The bilateral angular fractures included complex fractures of the vertical and horizontal mandible on the left side with interposition of bone fragments. The temporomandibular joints were fractured with medial displacement. The bilateral orbital fractures exhibited minor dislocation on the right side, while the floor and lateral wall were engaged on the left side. The zygomatic complex on the left side was displaced. The case before and after planning is shown in Fig. 6.

### Evaluation results and observations

The surgeon completed the reconstruction shown in Fig. 6 in 22 min after 45 min of training on the practice case. The fractures in the mandible are adequately reduced. It was not possible to obtain perfect occlusion due to inference from dislocated teeth; a tool to remove unwanted parts could be useful in such situations. The surgeon made extensive use of the grouping tool to build groups of fragments once he found a good fit. He also used the head tracking feature more and more throughout the session to look around objects instead of relying on rotation to get good visibility. He noted that he could perceive haptically when a bone fragment under manipulation did not fit due to misplacement, or due to inadequate reconstruction of previously positioned fragments. He also commented that the system is useful for understanding the complexity of the specific case, and that he during the planning process gained insights on preferred order of fragment placement; assembling the fragments in a certain order may provide valuable clues toward the best global reconstruction. The surgeon did not favor Snap-to-fit in this case after trying it on some fragments that were too small to give a robust result. The surgeon relied, therefore, on contact forces and visual inspection to complete the reconstruction.

## Discussion

Similar to the success of haptics in surgery training simulators [9–11], we believe that haptics can greatly improve the efficacy of CMF surgery planning software. In order to produce a complete planning tool, we need to add a number of features. A robust automatic method to find an initial segmentation complemented by interactive segmentation to remove unwanted objects during a planning session, such as the dislocated teeth in the evaluation case, would be of high value. Future work also includes the virtual design of reconstruction plates for additive production prior to surgery [21] and to explore ways to transfer the reconstruction plan to the operating room. There is also a need for a function which allows shaping and fitting of bone grafts or biomaterial to repair defects acquired from trauma. Finally, a thorough evaluation with several surgeons including a comparison with existing CMF planning software packages is needed to establish the efficacy of our system.

## Conclusions

We have described work in progress on a system that supports the planning of skeletal anatomy restoration in complex trauma cases. The key features are as follows: stereo graphics and head tracking that enables “look-around” to allow the user to graphically view the patient-specific anatomy in 3D from different angles by simply moving his/her head; stable six-DOF high-precision haptic rendering that provides intuitive guidance when manipulating virtual bone fragments, allowing the user to feel when one fragment is in contact with another; and Snap-to-fit that complements the contact forces with attraction forces to aid the precise placement of larger fragments such as the zygomatic bone. Grouping allows several bone fragments to be manipulated as one entity. Preliminary testing with one surgeon indicates that our haptic planning system has the potential to become a powerful tool that with little training allows a surgeon to complete a complex CMF surgery plan in a short amount of time.
